# Multivariable Models Incorporating Multiparametric Magnetic Resonance Imaging Efficiently Predict Results of Prostate Biopsy and Reduce Unnecessary Biopsy

**DOI:** 10.3389/fonc.2020.575261

**Published:** 2020-11-11

**Authors:** Shuanbao Yu, Guodong Hong, Jin Tao, Yan Shen, Junxiao Liu, Biao Dong, Yafeng Fan, Ziyao Li, Ali Zhu, Xuepei Zhang

**Affiliations:** ^1^ Department of Urology, The First Affiliated Hospital of Zhengzhou University, Zhengzhou, China; ^2^ Department of Nosocomial Infection Management, The First Affiliated Hospital of Zhengzhou University, Zhengzhou, China; ^3^ Key Laboratory of Precision Diagnosis and Treatment for Chronic Kidney Disease in Henan Province, Zhengzhou, China

**Keywords:** prostate cancer, prostate-specific antigen, prostate volume, multiparametric magnetic resonance imaging, prostate biopsy

## Abstract

**Purpose:**

We sought to develop diagnostic models incorporating mpMRI examination to identify PCa (Gleason score≥3+3) and CSPCa (Gleason score≥3+4) to reduce overdiagnosis and overtreatment.

**Methods:**

We retrospectively identified 784 patients according to inclusion criteria between 2016 and 2020. The cohort was split into a training cohort of 548 (70%) patients and a validation cohort of 236 (30%) patients. Age, PSA derivatives, prostate volume, and mpMRI parameters were assessed as predictors for PCa and CSPCa. The multivariable models based on clinical parameters were evaluated using area under the curve (AUC), calibration plots, and decision curve analysis (DCA).

**Results:**

Univariate analysis showed that age, tPSA, PSAD, prostate volume, MRI-PCa, MRI-seminal vesicle invasion, and MRI-lymph node invasion were significant predictors for both PCa and CSPCa (each *p*≤0.001). PSAD has the highest diagnostic accuracy in predicting PCa (AUC=0.79) and CSPCa (AUC=0.79). The multivariable models for PCa (AUC=0.92, 95% CI: 0.88–0.96) and CSPCa (AUC=0.95, 95% CI: 0.92–0.97) were significantly higher than the combination of derivatives for PSA (*p*=0.041 and 0.009 for PCa and CSPCa, respectively) or mpMRI (each *p*<0.001) in diagnostic accuracy. And the multivariable models for PCa and CSPCa illustrated better calibration and substantial improvement in DCA at threshold above 10%, compared with PSA or mpMRI derivatives. The PCa model with a 30% cutoff or CSPCa model with a 20% cutoff could spare the number of biopsies by 53%, and avoid the number of benign biopsies over 80%, while keeping a 95% sensitivity for detecting CSPCa.

**Conclusion:**

Our multivariable models could reduce unnecessary biopsy without comprising the ability to diagnose CSPCa. Further prospective validation is required.

## Introduction

Prostate cancer (PCa) is the most common malignancy of the male reproductive system, and the fifth leading cause of cancer death among men worldwide, with over one million new cases and 358,989 deaths in 2018 (1, 2). Although the incidence of PCa in China is relatively low compared with western countries, it has been progressively rising due to the aging population, changing diets, and availability of medical screening in recent decades (3–5).

A dozen of nomograms for prediction of PCa and/or clinically significant prostate cancer (CSPCa) with Gleason score (GS) ≥3+4 had been developed in Western countries (6–9). Researchers found that the models derived from Western populations overestimated the predicated risk of PCa and CSPCa among the East Asian populations, mainly due to the racial differences between two populations (10, 11). It may indicate the essentiality of developing risk prediction models among Chinese and Asian population. Additionally, the predict models for PCa and CSPCa among Chinese populations were mostly based on age, prostate-specific antigen (PSA) derivatives, prostate volume (PV), transrectal ultrasound (TRUS) finding, and digital rectal examination in the current studies (12–15).

Studies have validated the clinical utility of multiparametric magnetic resonance imaging (mpMRI) for the detection and localization of International Society of Urological Pathology (ISUP) grade ≥2 cancers (16), and demonstrated that the mpMRI may help mitigate the racial disparities of PCa (17). However, as far as we know, the knowledge about the performance of risk prediction models incorporating mpMRI findings is limited. In our study, we evaluated the diagnostic accuracy of age, PSA derivatives (tPSA, f/tPSA, and PSAD), PV, and mpMRI parameters for predicting PCa and CSPCa, respectively. Additionally, multivariable models based on age, PSA derivates, PV, and mpMRI parameters were developed to predict PCa and CSPCa. Overall, this study will be useful for developing the Chinese and international multivariable model based on clinical parameters to diagnose PCa and CSPCa, thereby reducing unnecessary prostate biopsy, and selecting the best clinical strategy.

## Materials and Methods

### Study Populations

**Figure 1:**
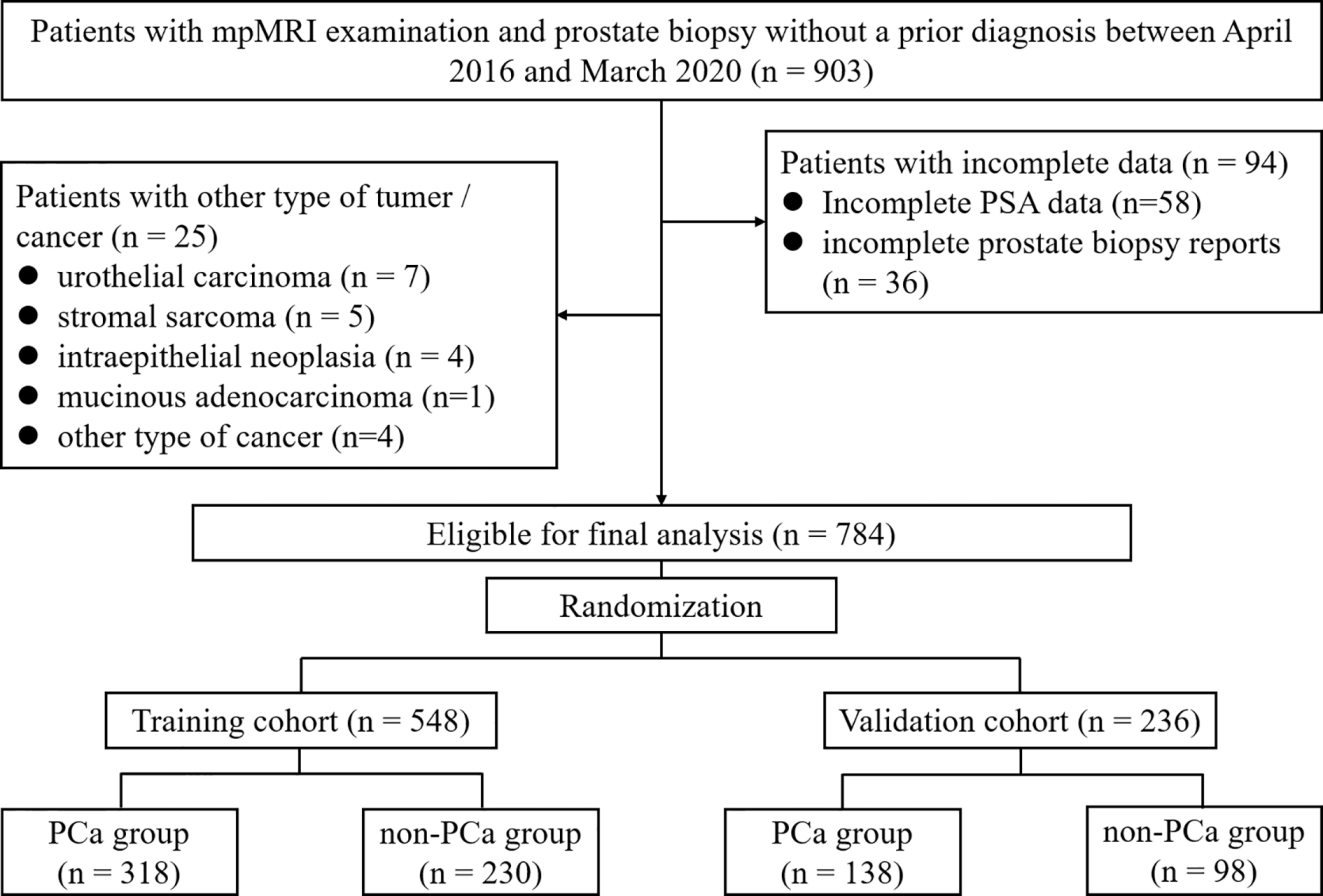
Flowchart of study participants selection.

This retrospective study was approved by the Institutional Ethics Review Board, and a waiver of informed consent was obtained. Between April 2016 and March 2020, mpMRI examination and TRUS-guided prostate biopsy was performed among 903 consecutive patients without a prior PCa diagnosis. The 25 patients diagnosed with other type of tumor/cancer and 94 patients with incomplete data were excluded leaving 784 cases available for analysis (**Figure 1**).

### Clinical, Imaging, and Pathological Parameters Collection

The clinical variables including the age at prostate biopsy, serum tPSA and fPSA level, PV, reports of mpMRI examination, and results of prostate biopsy were extracted from clinical records. PV was measured by 3.0-T MRI system (Siemens, Germany) using the exact prolate ellipsoid formula: PV = transversal diameter × anteroposterior diameter × vertical diameter × π/6 (18). The f/tPSA was calculated by dividing fPSA by tPSA, and the PSAD was calculated by dividing tPSA by PV.

All mpMRI examinations were performed using the 3.0-T MRI system with a pelvic phased-array coil, complied with European Society of Urology Radiology guidelines. The scan protocol for all patients included T2-weighted imaging (T2WI), diffusion-weighted imaging (DWI) and dynamic contrast-enhanced imaging (DCE). Additionally, 513 patients included the magnetic resonance spectroscopic imaging (MRSI). The prostate mpMRI images were interpreted by two experienced genitourinary radiologists with at least three years of prostate mpMRI experience. The mpMRI results were divided into groups according to the reports: “negative”, “equivocal”, and “suspicious” for the presence of PCa (MRI-PCa), seminal vesicle invasion (MRI-SVI), and lymph node invasion (MRI-LNI). The “negative”, “equivocal”, and “suspicious” for MRI-PCa corresponded to the PI-RADS 1 or 2, PI-RADS 3, and PI-RADS 4 or 5 according to the latest Prostate Imaging Reporting and Data System version 2 (PI-RADS v2) guideline. The suspicious MRI-SVI was defined as loss of normal high signal within and/or along the seminal vesicles (19). The suspicious MRI-LNI was defined as lymph nodes >8 mm in short-axis dimension and those with a high signal intensity on DWI (20).

All patients underwent TRUS-guided systematic 12-point biopsy according to the same protocol by three surgeons. If suspected malignant nodules by mpMRI and/or ultrasound, additional 1–5 needles were performed in regions with cognitive MRI-TURS fusion and/or abnormal ultrasound echoes. Biopsy cores were analyzed according to the standards of ISUP (21).

### Statistical Analysis

We described the profile of age, PSA derivatives (tPSA, f/tPSA, PSAD), PV, and mpMRI parameters (MRI-PCa, MRI-SVI, and MRI-LNI) of enrolled patients by pathological diagnosis. Student’s t test or ANOVA was used to analyze continuous data. The Mann-Whitney U test was used to analyze ranked data. The χ^2^ test or Fisher’s exact test was used to analyze categorical data. The Bonferroni method for multiple comparisons was used if significant difference between groups was noted. The univariable and multivariable logistic regression were performed to identify significant predictors of PCa and CSPCa on biopsy. The models were examined with the receiver operating characteristics (ROC) curve and calibration plots, and the clinical utility was evaluated with a decision-curve analysis (DCA) (22). The ROC curve, calibration plots, and DCA was constructed with the package of “plotROC”, “ggplot2”, and “rmda”. Differences between the area under the curve (AUC) were compared using the method of DeLong et al. The calibration was assessed by grouping men in the validation cohort into delices (each of size 23 or 24), and then comparing the mean of predicated probabilities and the observed proportions. The sum squares of the residues (SSR) was used to assess the deviation of calibration plots form the 45° line. All tests were two sided with significance level set at 0.05. Data cleaning and analyses were conducted using R statistical software (Version 3.6.2).

## Results

A total of 784 cases met study inclusion criteria. The training and validation cohorts consisted of 548 (70%) and 236 (30%) men, respectively (**Figure 1** and **Supplementary Table 1**). The patient characteristics are summarized in **Supplementary Table 1**. The median [interquartile range (IQR)] age, tPSA, f/tPSA, PSAD, and PV values were 68 (62–74) years, 14.7 (8.22–29.0) ng/ml, 0.13 (0.09–0.20), 0.28 (0.15–0.61) ng/ml^2^, and 51 (34–74) ml, respectively. The MRI-PCa, -SVI, and -LNI were classified as “suspicious” in 355 (45%), 118 (15%), and 32 (4%) patients, respectively. Prostate biopsy results were negative for 457 (58%) cases and positive for 327 (42%) cases. Of the 327 PCa patients, 46 (14%) were with GS≤3+3, 50 (15%) were with GS=3+4, 88 (27%) were with GS=4+3, and 143 (44%) were with GS≥4+4 (**Supplementary Table 1**).

### The Clinical Characteristics of Enrolled Patients by Pathological Results

**Table 1** listed clinical characteristics including mpMRI parameters of enrolled patients by GS. The age (70 vs 66 years, *p*<0.001), tPSA (26.5 vs 10.9 ng/ml, *p*<0.001), and PSAD (0.62 vs 0.19 ng/ml^2^, *p*<0.001) were significantly higher in PCa patients compared with no-PCa (**Supplementary Table 2**). Additionally, the concentration of tPSA increased in serum with the GS (**Table 1**). The f/tPSA (0.11 vs 0.15, *p*<0.001) and PV (41 vs 59 ml, *p*<0.001) was smaller in CSPCa compared with patients without CSPCa (**Table 1**). However, the f/tPSA and PV increased with GS among CSPCa patients (**Table 1**). As the GS increased, the proportions for suspicious presence of PCa, SV1, and LNI by mpMRI examination also increased (**Table 1**).

**Table 1:** The clinical characteristics of enrolled patients by Gleason score between April 2016 and March 2020.

Clinical characteristics	No-PCa (n=457)	GS≤3+3 (n=46)	GS=3+4 (n=50)	GS=4+3 (n=88)	GS≥4+4 (n=143)	*p*
Age (years)	66 (61–72)	70 (65–76)	70 (63–76)	70 (64–75)	70 (65–75)	<0.001
tPSA (ng/ml)	10.9 (6.68–17.3)	14.5 (7.88–21.8)	22.3 (13.0–36.0)	32.0 (15.6–59.8)	34.6 (19.8–56.4)	<0.001
f/tPSA	0.15 (0.10–0.21)	0.14 (0.10–0.18)	0.10 (0.07–0.13)	0.11 (0.07–0.16)	0.12 (0.07–0.18)	<0.001
PSAD (ng/ml^2^)	0.19 (0.12–0.31)	0.28 (0.16–0.54)	0.61 (0.39–0.90)	0.81 (0.52–1.46)	0.65 (0.33–1.27)	<0.001
PV (ml)	59 (40–84)	47 (30–71)	37 (25–59)	38 (28–51)	42 (32–64)	<0.001
MRI-PCa, No. (%)						<0.001
Negative	254 (56)	14 (30)	12 (24)	9 (10)	7 (5)	
Equivocal	99 (22)	9 (20)	10 (20)	4 (5)	11 (8)	
Suspicious	104 (23)	23 (50)	28 (56)	75 (85)	125 (87)	
MRI-SVI, No. (%)						<0.001_*_
Negative	453 (99)	42 (91)	42 (84)	54 (61)	62 (43)	
Equivocal	1 (0.2)	0 (0)	1 (2)	6 (7)	5 (3)	
Suspicious	3 (0.7)	4 (9)	7 (14)	28 (32)	76 (53)	
MRI-LNI, No. (%)						<0.001^*^
Negative	446 (98)	44 (96)	47 (94)	72 (82)	96 (67)	
Equivocal	11 (2)	1 (2)	0 (0)	10 (11)	25 (17)	
Suspicious	0 (0)	1 (2)	3 (6)	6 (7)	22 (15)	

PCa, prostate cancer; GS, Gleason score; tPSA, total prostate-specific antigen; f/tPSA, free PSA/total PSA; PV, prostate volume; SVI, seminal vesicle invasion; LNI, lymph node invasion. *Due to small number for equivocal of MRI-SVI and MRI-LNI, when calculating the p value, the equivocal group was merged into the suspicious group of MRI-SVI and MRI-LNI, respectively.

### Univariable Analysis of Clinical Parameters for Predicting PCa and CSPCa

In univariable logistic regression analysis, all clinical variables excepting f/tPSA were significant predictors for PCa and CSPCa (each *p*≤0.001, **Table 2**). Regarding PSA derivatives, the risk of PCa and CSPCa increased with tPSA (OR=1.05 for both PCa and CSPCa) and PSAD (OR=3.27 for PCa, and OR=19.6 for CSPCa). PSAD performed best in predicting PCa (AUC=0.79, 95% CI: 0.75–0.83) and CSPCa (AUC=0.79, 95% CI: 0.75–0.83) (**Table 2**). The risk of PCa (OR=0.99, 95% CI: 0.98–0.99) and CSPCa (OR=0.98, 95% CI: 0.98–0.99) was negatively associated with prostate volume, which displayed relatively low diagnostic accuracy in prediction of PCa (AUC=0.65, 95% CI: 0.61–0.70) and CSPCa (AUC=0.65, 95% CI: 0.61–0.70). Regarding mpMRI examination, MRI-PCa achieved the highest diagnostic accuracy in prediction of PCa (AUC=0.78, 95% CI: 0.74–0.82) and CSPCa (AUC=0.78, 95% CI: 0.74–0.82) (**Table 2**). The combination of PSA derivatives (AUC=0.88 for PCa, and AUC=0.90 for CSPCa) or mpMRI derivatives (AUC=0.84 for PCa, and AUC=0.86 for CSPCa) outperformed single derivatives in diagnostic of PCa and CSPCa (all *p*<0.05).

**Table 2:** Univariable regression analysis of clinical parameters to predict PCa and CSPCa.

Clinical parameter	PCa (GS≥3+3)			CSPCa (GS≥3+4)		
	OR (95% CI)	AUC (95% CI)	*p*	OR (95% CI)	AUC (95% CI)	*p*
Age (years)	1.04 (1.02–1.06)	0.60 (0.55–0.65)	<0.001	1.03 (1.01–1.05)	0.60 (0.55–0.65)	0.001
tPSA (ng/ml)	1.05 (1.04–1.06)	0.73 (0.69–0.78)	<0.001	1.05 (1.04–1.06)	0.73 (0.69–0.78)	<0.001
f/tPSA	1.13 (0.67–1.90)	0.61 (0.56–0.66)	0.644	1.21 (0.71–2.05)	0.61 (0.56–0.66)	0.489
PSAD (ng/ml^2^)*	3.27 (2.59–4.14)	0.79 (0.75–0.83)	<0.001	19.6 (10.5–36.4)	0.79 (0.75–0.83)	<0.001
PV (ml)	0.99 (0.98–0.99)	0.65 (0.61–0.70)	<0.001	0.98 (0.98–0.99)	0.65 (0.61–0.70)	<0.001
MRI-PCa (negative as reference)						
Equivocal	2.54 (1.41–4.58)		0.002	2.47 (1.26–4.85)		0.009
		0.78 (0.74–0.82)			0.78 (0.74–0.82)	
Suspicious	14.0 (8.69–22.6)		<0.001	15.4 (9.09–26.1)		<0.001
MRI-SVI	97.9 (23.8–403)	0.69 (0.66–0.72)	<0.001	53.7 (21.3–135)	0.69 (0.66–0.72)	<0.001
MRI-LNI	13.0 (5.45–31.0)	0.59 (0.56–0.62)	<0.001	14.8 (6.54–33.6)	0.59 (0.56–0.62)	<0.001

*Parameter was log-transformed; PCa, prostate cancer; CSPCa, clinically significant prostate cancer; GS, Gleason score; tPSA, total prostate-specific antigen; f/tPSA, free PSA/total PSA; PSAD, prostate-specific antigen density; PV, prostate volume; SVI, seminal vesicle invasion; LNI, lymph node invasion.

### Development of Multivariable Models Incorporating PSA Derivates, Prostate Volume, and mpMRI Parameters

**Figure 2:**
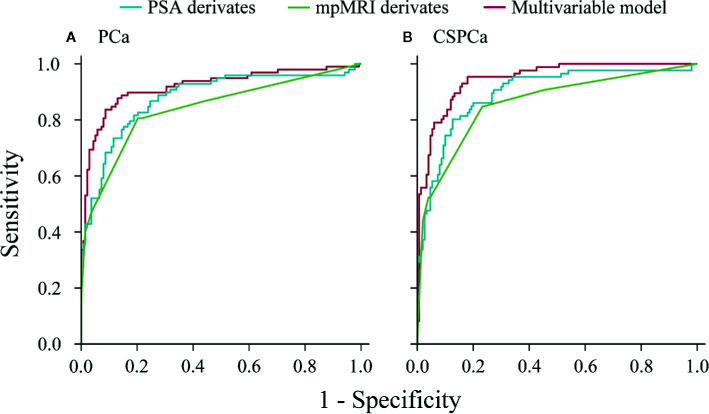
Receive operating characteristic curves of PSA derivatives, mpMRI derivatives, and multivariable models for predicting prostate cancer and clinically significant prostate cancer in the validation cohort. **(A)** PCa: Gleason score≥3+3, **(B)** CSPCa: Gleason score≥3+4.

**Figure 3:**
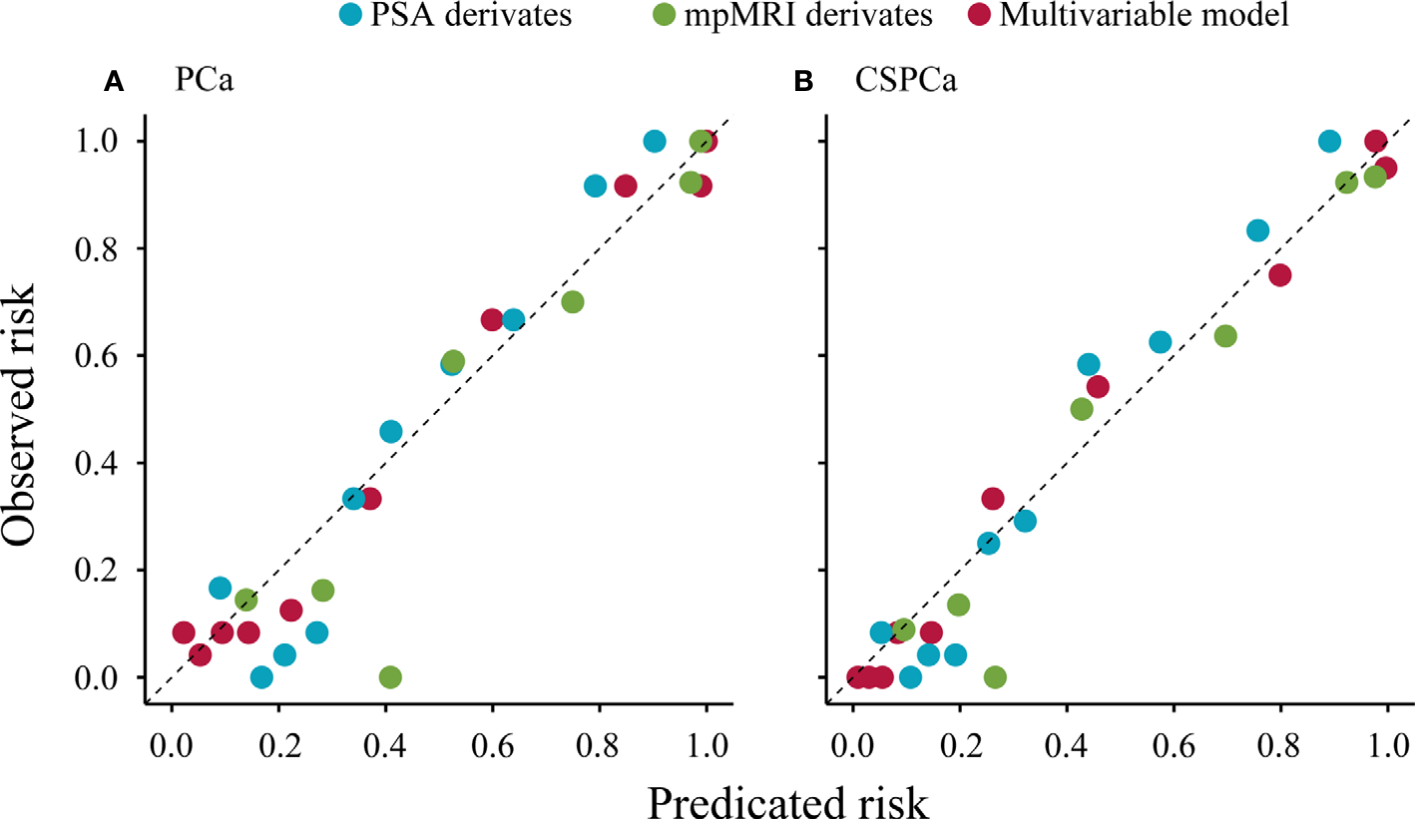
Calibration plot of observed vs predicted rick of prostate cancer and clinically significant prostate cancer using PSA derivatives, mpMRI derivatives, and multivariable models in the validation cohort. **(A)** PCa: Gleason score≥3+3, **(B)** CSPCa: Gleason score≥3+4.

In a stepwise multivariable analysis, age, tPSA, PV, MRI-PCa, and MRI-SVI reminded in the model for detection of PCa (each *p*≤0.001). The multivariable model for CSPCa was established including age (*p*=0.002), tPSA (*p*<0.001), PV (*p*<0.001), MRI-PCa (*p*<0.001), MRI-SVI (*p*<0.001), and MRI-LNI (*p*<0.001) (**Table 3**). The multivariable models for PCa (AUC=0.92, 95%CI: 0.88–0.96) and CSPCa (AUC=0.95, 95%CI: 0.92–0.97) were significantly higher than the combination of derivates for PSA (*p*=0.041 and 0.009 for PCa and CSPCa, respectively) or mpMRI examination (each *p*<0.001) in diagnostic accuracy (**Figure 2**). The calibration plots of predicated risk against observed PCa and CSPCa risk indicated excellent concordance in multivariable models (SSR=0.033 for PCa, and SSR=0.025 for CSPCa), followed by PSA derivatives (SSR=0.130 for PCa, and SSR=0.086 for CSPCa) and mpMRI derivatives (SSR=0.190 for PCa, and SSR=0.083 for CSPCa) (**Figure 3**).

**Table 3:** Multivariable regression analysis of clinical parameters to predict PCa and CSPCa.

Clinical parameter	PCa (GS≥3+3)			CSPCa (GS≥3+4)		
	Coefficient	OR (95% CI)	*p*	Coefficient	OR (95% CI)	*p*
Intercept	-5.018	NA	<0.001	-4.336	NA	<0.001
Age (years)	0.060	1.06 (1.03–1.09)	<0.001	0.045	1.05 (1.02–1.08)	0.002
tPSA (ng/ml)	0.050	1.05 (1.03–1.07)	<0.001	0.053	1.05 (1.04–1.07)	<0.001
PV (ml)	-0.031	0.97 (0.96–0.98)	<0.001	-0.037	0.96 (0.95–0.97)	<0.001
MRI-PCa (negative as reference)						
Equivocal	0.650	1.92 (0.98–3.74)	0.057	0.535	1.71 (0.78–3.73)	0.180
Suspicious	1.693	5.44 (3.10–9.54)	<0.001	1.635	5.13 (2.71–9.70)	<0.001
MRI-SVI	5.055	156 (17.9–1396)	<0.001	3.546	34.7 (8.53–140)	<0.001
MRI-LNI	NA	NA	NA	1.429	4.18 (1.28–13.6)	<0.001

PCa, prostate cancer; CSPCa, clinically significant prostate cancer; GS, Gleason score; tPSA, total prostate-specific antigen; PV, prostate volume; SVI, seminal vesicle invasion; LNI, lymph node invasion; NA, not applicable.

### Impact of Multivariable Model on Biopsies Averted and CSPCa Diagnosis Delayed

**Figure 4:**
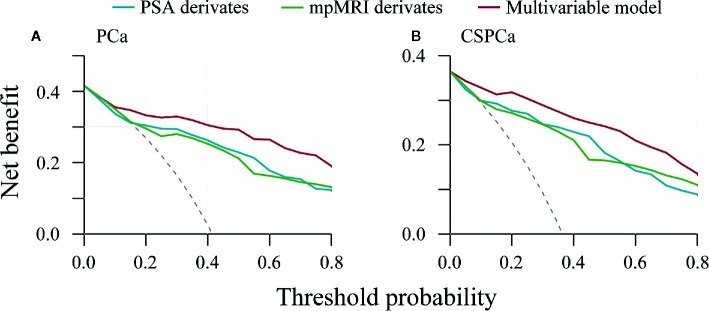
Decision curve analysis of PSA derivatives, mpMRI derivatives, and multivariable models for predicting prostate cancer and clinically significant prostate cancer in the validation cohort. **(A)** PCa: Gleason score≥3+3, **(B)** CSPCa: Gleason score≥3+4.

To further assess potential clinical benefit of the multivariable models, we performed DCA using the predicted risk in the validation cohort. It was observed that the multivariable model for PCa had the highest net benefit across the threshold probabilities above 10% (**Figure 4A**), and the CSPCa model had the highest clinical benefit across a range of relevant threshold probabilities, compared with the combination of PSA derivatives or mpMRI derivatives (**Figure 4B**). Clinical consequences of using various cut-offs for PSA derivatives, mpMRI derivatives, and multivariable models (compared with the strategy of biopsy all patients), including the number of biopsies that could be avoided and the number of PCa by GS that would be missed was displayed in **Table 4**. Using of a 30% cutoff for the PCa model would allow for sparing 124/236 (53%) of prostate biopsy, avoiding 114/138 (83%) of benign biopsies and reducing 6/12 (50%) of low-risk PCa diagnosis at the cost of delaying 4/86 (5%) of CSPCa (**Table 4**). At the same level of sensitivity as the PCa model to detect CSPCa, CSPCa model with a threshold of 20% could spare the number of biopsies by 126/236 (53%), avoid the number of benign biopsies by 116/138 (84%), and reduce the number of low-risk PCa diagnosis by 6/12 (50%) (**Table 4**).

**Table 4:** Impact of using PSA derivates, mpMRI derivates, and multivariable model on biopsies avoided or delayed.

Models	Cut-offf or predicted risk	Sensitivity for detecting CSPCa	Specificity for detecting CSPCa	Biopsies avoided(n=236), n (%)	Delayed			
					GS=3+3(n=12), n (%)	GS=3+4(n=12), n (%)	GS=4+3(n=27), n (%)	GS≥4+4(n=47), n (%)
PCa								
PSA derivates	10%	98%	7%	12 (5)	2 (17)	0 (0)	0 (0)	2 (4)
mpMRI derivates	10%	100%	0%	0 (0)	0 (0)	0 (0)	0 (0)	0 (0)
Multivariate model	10%	99%	40%	61 (26)	4 (33)	0 (0)	1 (4)	0 (0)
PSA derivates	20%	98%	34%	53 (22)	2 (17)	0 (0)	0 (0)	2 (4)
mpMRI derivates	20%	91%	55%	90 (38)	5 (42)	3 (25)	3 (11)	2 (4)
Multivariate model	20%	97%	65%	100 (42)	5 (42)	1 (8)	1 (4)	1 (2)
PSA derivates	30%	95%	63%	98 (42)	4 (33)	0 (0)	1 (4)	3 (6)
mpMRI derivates	30%	85%	76%	127 (54)	6 (50)	5 (42)	3 (11)	5 (11)
Multivariate model	30%	95%	80%	124 (53)	6 (50)	2 (17)	1 (4)	1 (2)
PSA derivates	40%	86%	79%	130 (55)	6 (50)	2 (17)	3 (11)	7 (15)
mpMRI derivates	40%	85%	77%	128 (54)	6 (50)	5 (42)	3 (11)	5 (11)
Multivariate model	40%	91%	84%	134 (57)	6 (50)	4 (33)	1 (4)	3 (6)
CSPCa								
PSA derivates	5%	98%	5%	9 (4)	1 (8)	0 (0)	0 (0)	2 (4)
mpMRI derivates	5%	100%	0%	0 (0)	0 (0)	0 (0)	0 (0)	0 (0)
Multivariate model	5%	100%	36%	54 (23)	3 (25)	0 (0)	0 (0)	0 (0)
PSA derivates	10%	98%	19%	31 (13)	2 (17)	0 (0)	0 (0)	2 (4)
mpMRI derivates	10%	91%	55%	90 (38)	5 (42)	3 (25)	3 (11)	2 (4)
Multivariate model	10%	98%	61%	93 (39)	5 (42)	1 (8)	1 (4)	0 (0)
PSA derivates	15%	93%	41%	67 (28)	2 (17)	2 (17)	2 (7)	2 (4)
mpMRI derivates	15%	91%	55%	90 (38)	5 (42)	3 (25)	3 (11)	2 (4)
Multivariate model	15%	95%	69%	108 (46)	6 (50)	2 (17)	1 (4)	1 (2)
PSA derivates	20%	95%	55%	87 (37)	3 (25)	0 (0)	1 (4)	3 (6)
mpMRI derivates	20%	85%	76%	127 (54)	6 (50)	5 (42)	3 (11)	5 (11)
Multivariate model	20%	95%	81%	126 (53)	6 (50)	2 (17)	1 (4)	1 (2)

PSA derivates include tPSA, f/tPSA, and PSAD; mpMRI derivates include MRI-PCa, MRI-SVI, and MRI-SVI; Multivariable model for PCa includes age, tPSA, PV, MRI-PCa, and MRI-SVI; Multivariable model for CSPCa includes age, tPSA, PV, MRI-PCa, MRI-SVI, and MRI-LNI; GS, Gleason score; PCa, prostate cancer; CSPCa, clinically significant prostate cancer.

## Discussion

In our study, we assessed the performance of age, PSA derivatives, PV, and mpMRI parameters in diagnostic of PCa and CSPCa. This study revealed that age, tPSA, PSAD, PV, MRI-PCa, MRI-SVI, and MRI-LNI were significant predictors for both PCa and CSPCa. Additionally, we developed multivariable models based on clinical parameters including mpMRI derivatives, which outperformed the combination of PSA or mpMRI derivatives in diagnostic of PCa and CSPCa. Use the multivariable PCa model with a cutoff of 30% or CSPCa model with a cutoff of 20% could spare the number of prostate biopsies by 53%, avoid the number of benign biopsies over 80%, and reduce the number of low-risk PCa diagnosis by 50%. Importantly, this can be achieved without compromising the ability to detection of CSPCa.

In this study, we analyzed the relationship between clinical characteristics and GS, and found the non-linear pattern between f/tPSA and PV, and GS. This may explain the inconsistent performance of f/tPSA in detecting of PCa (23, 24), and the relatively low diagnostic accuracy of PV in prediction of PCa and CSPCa (25). PSAD performed best in prediction of PCa and CSPCa among PSA derivates in our study. However, the stepwise multivariable models included tPSA and PV, rather than the PSAD. This may suggest that fitting independent variable individually rather than the PSAD was superior in constructing multivariable models (26). The DRE was excluded as a risk factor because of potential interobserver variability in its assessment (7).

The mpMRI improved the detection of CSPCa due to its anatomic detail, emerging accessibility, and addition of functional data. A growing body of literatures has validated the clinical utility of mpMRI in the detection and localization of CSPCa (16, 27, 28). In our study, the MRI-PCa also had the highest performance in PCa (AUC=0.78) and CSPCa (AUC=0.78) detection among mpMRI parameters. Additionally, we found that the combination of mpMRI parameters including MRI-PCa, MRI-SVI, and MRI-LNI could enhance the diagnostic accuracy in prediction of PCa (AUC=0.84) and CSPCa (AUC=0.86) compared with single mpMRI parameter. Moreover, mpMRI radiomics features significantly associated with PCa aggressiveness on the histopathological and genomics levels (29, 30). And mpMRI parameters including MRI-extracapsular extension (ECE), -SVI, -LNI had been recognized as significant predictors of LNI (20). These may suggest that addition of objective mpMRI parameters could increase the performance, and reduce the inter-reader (31) and inter-center variability (32) of PI-RADS v2 for PCa and CSPCa diagnosis.

Furthermore, we developed multivariable models, which outperformed PSA and mpMRI derivatives in prediction of PCa and CSPCa. Using a PCa risk threshold of 30% or CSPCa risk threshold of 20% would spare 53% of prostate biopsies and avoid over 80% of benign biopsies at the cost of missing 5% of CSPCa. Although cross-study comparisons are challenging, the multivariable models based on clinical parameters performed better than Huashan risk calculators (AUC=0.85 and 0.86 for PCa and CSPCa, respectively) (13), CRCC-PC (AUC=0.80 and 0.83 for PCa and CSPCa, respectively) (15) and MRI-ERSPC-RC (AUC=0.85 for CSPCa) (6). However, our results compared unfavorably to those Risk calculators incorporating novel markers, including 4Kscore-ERSPC (reduced biopsies by 66% at the cost of missing 2% of CSPCa) (33), PCA3-based nomogram (reduced biopsies by 55% at the cost of missing 2% of CSPCa) (8), and MiPS-PCPT RC (reduced biopsies by 47% at the cost of missing 2% of CSPCa) (34). These differences further demonstrate that the novel molecular biomarkers add value in detection of PCa and CSPCa. In the future, the multivariable models combining molecular biomarkers, mpMRI parameters, and clinical parameters should be developed to better identify PCa and CSPCa, and avoid unnecessary prostate biopsy and overtreatment. Overall, our study provided basis for developing the model based on clinical parameter including mpMRI parameters to diagnosis PCa and CSPCa among Chinese population.

Our study was subject to several limitations. First, this study was a single center study and limited by the inherent drawbacks of its retrospective design. Second, the PI-RADS v2 scores were not used in our study and no central review of mpMRI examination was present. However, the combination of mpMRI parameters including MRI-PCa, -SVI, and -LNI performed similar with PI-RADS v2 in diagnostic of PCa (AUC=0.84 vs 0.83–0.86) (35, 36), and CSPCa (AUC=0.86 vs 0.87–0.91) (35, 37). Third, we acknowledge that the inclusion of new biomarkers, for example, prostate cancer susceptibility loci, 4K score, prostate cancer gene 3, and other genomic markers may strengthen our diagnostic models and may be considered for future studies. However, the advantage of our model is its simplicity and cheapness, which could facilitate its implementation in clinical practice.

## Conclusions

Our study found the non-linear pattern between f/tPSA and PV, and GS, and demonstrated that age, tPSA, PSAD, PV, and mpMRI parameters were significant predictors for both PCa and CSPCa. The multivariable model for PCa with a 30% cutoff or the CSPCa model with a 20% cutoff, could spare the number of unnecessary biopsies by 53%, avoid the number of benign biopsies over 80%, and reduce the number of low-risk PCa diagnosis by 50%, while missing only a minimal number (5%) of CSPCa. Further prospective validation is required.

## Data Availability Statement

The raw data supporting the conclusions of this article will be made available by the authors, without undue reservation.

## Ethics Statement

The studies involving human participants were reviewed and approved by Ethics Review Committee of life sciences, Zhengzhou University. Written informed consent for participation was not required for this study in accordance with the national legislation and the institutional requirements.

## Author Contributions

XZ and SY conceptualized, designed, and supervised the study. GH, SY, JT, YS, JL, BD, YF, ZL, and AZ coordinated and participated data collection. SY and YS carried out the statistical analysis and drafted the manuscript. XZ and JT provided guidance on data analysis. XZ and YS revised the manuscript. All authors contributed to the article and approved the submitted version.

## Funding

This work was supported by grants from the Henan Medical Science and Technology Project [grant no. LHGJ20190181] and the Henan Science and Technology Project [grant No. 162102310204].

## Conflict of Interest

The authors declare that the research was conducted in the absence of any commercial or financial relationships that could be construed as a potential conflict of interest.
