# Supplementary material for: Multivariable Models Incorporating Multiparametric Magnetic Resonance Imaging Efficiently Predict Results of Prostate Biopsy and Reduce Unnecessary Biopsy
Source: Front Oncol. 2020 Nov 11;10:575261. doi: 10.3389/fonc.2020.575261 (PMC7688051; doi:10.3389/fonc.2020.575261)
Supplement: Supplementary file 1 [file DataSheet_1.docx]

Supplementary Material

**Supplementary Table 1 |** The clinical characteristics for all enrolled patients, training cohort, and validation cohort

| Clinical characteristics | All enrolled participants  (n=784) | Training cohort  (n=548) | Validation cohort  (n=236) |
| --- | --- | --- | --- |
| Age (years) | 68 (62-74) | 68 (62-74) | 68 (62-73) |
| tPSA (ng/ml) | 14.7 (8.22-29.0) | 14.8 (7.97-28.6) | 14.2 (8.82-29.5) |
| f/tPSA | 0.13 (0.09-0.20) | 0.13 (0.09-0.20) | 0.13 (0.09-0.19) |
| PSAD (ng/ml^2^) | 0.28 (0.15-0.61) | 0.28 (0.15-0.59) | 0.28 (0.15-0.63) |
| PV (ml) | 51 (34-74) | 50 (34-73) | 53 (35-74) |
| MRI-PCa, No. (%) | |  |  |
| Negative | 296 (38) | 205 (37) | 91 (39) |
| Equivocal | 133 (17) | 95 (17) | 38 (16) |
| Suspicious | 355 (45) | 248 (45) | 107 (45) |
| MRI-SVI, No. (%) | |  |  |
| Negative | 653 (83) | 458 (84) | 195 (83) |
| Equivocal | 13 (2) | 10 (2) | 3 (1) |
| Suspicious | 118 (15) | 80 (15) | 38 (16) |
| MRI-LNI, No. (%) | |  |  |
| Negative | 705 (90) | 496 (91) | 209 (89) |
| Equivocal | 47 (6) | 27 (5) | 20 (8) |
| Suspicious | 32 (4) | 25 (5) | 7 (3) |
| Biopsy result |  |  |  |
| No-PCa | 457 (58) | 319 (58) | 138 (58) |
| GS≤3+3 | 46 (6) | 34 (6) | 12 (5) |
| GS=3+4 | 50 (6) | 38 (7) | 12 (5) |
| GS=4+3 | 88 (11) | 61 (11) | 27 (11) |
| GS≥8 | 143 (18) | 96 (18) | 47 (20) |

PCa: prostate cancer; GS: Gleason score; tPSA: total prostate-specific antigen; f/tPSA: free PSA / total PSA; PV: prostate volume; SVI: seminal vesicle invasion; LNI: lymph node invasion. *: Due to small number for equivocal of MRI-SVI and MRI-LNI, when calculating the *p* value, the equivocal group was merged into the suspicious group of MRI-SVI and MRI-LNI, respectively.

**Supplementary Table 2 |** The clinical characteristics of enrolled patients stratified by pathological results

| Clinical characteristics | PCa (GS≥3+3) | | |  | CSPCa (GS≥3+4) | | |
| --- | --- | --- | --- | --- | --- | --- | --- |
|  | No (n=456) ^*^ | Yes (n=328) ^*^ | *p* |  | No (n=503) ^*^ | Yes (n=281) ^*^ | *p* |
| Age (years) | 66 (61-72) | 70 (64-75) | <0.001 |  | 67 (61-72) | 70 (64-75) | <0.001 |
| tPSA (ng/ml) | 10.9 (6.68-17.3) | 26.5 (13.6-50.1) | <0.001 |  | 11.3 (6.80-18.0) | 30.1 (14.6-53.9) | <0.001 |
| f/tPSA | 0.15 (0.10-0.21) | 0.11 (0.07-0.17) | <0.001 |  | 0.14 (0.10-0.21) | 0.11 (0.07-0.16) | <0.001 |
| PSAD (ng/ml^2^) | 0.19 (0.11-0.31) | 0.62 (0.30-1.21) | <0.001 |  | 0.19 (0.12-0.32) | 0.66 (0.36-1.24) | <0.001 |
| PV (ml) | 59 (40-84) | 41 (29-60) | <0.001 |  | 58 (39-84) | 40 (29-59) | <0.001 |
| MRI-PCa, No. (%) |  |  | <0.001 |  |  |  | <0.001 |
| Negative | 254 (56) | 42 (13) |  |  | 268 (53) | 28 (10) |  |
| Equivocal | 99 (22) | 34 (10) |  |  | 108 (21) | 25 (9) |  |
| Suspicious | 103 (23) | 252 (77) |  |  | 127 (25) | 228 (81) |  |
| MRI-SVI, No. (%) |  |  | <0.001 |  |  |  | <0.001 |
| Negative | 452 (99) | 201 (61) |  |  | 495 (98) | 158 (56) |  |
| Suspicious ^#^ | 4 (1) | 127 (39) |  |  | 8 (2) | 123 (44) |  |
| MRI-LNI, No. (%) |  |  | <0.001 |  |  |  | <0.001 |
| Negative | 445 (98) | 260 (79) |  |  | 490 (97) | 215 (77) |  |
| Suspicious ^#^ | 11 (2) | 68 (21) |  |  | 13 (3) | 66 (23) |  |

^*^: Data are presented as median (interquartile range) unless other indicated; PCa: prostate cancer; CSPCa: clinically significant prostate cancer; tPSA: total prostate-specific antigen; f/tPSA: free PSA / total PSA; PSAD: prostate-specific antigen density; PV: prostate volume; SVI: seminal vesicle invasion; LNI: lymph node invasion; ^#^: Due to small number of equivocal group, which was merged into the suspicious group of MRI-SVI and MRI-LNI, respectively.
